# The versatile role of Serpina3c in physiological and pathological processes: a review of recent studies

**DOI:** 10.3389/fendo.2023.1189007

**Published:** 2023-05-23

**Authors:** Yang Li, Liang Guo

**Affiliations:** School of Exercise and Health and Shanghai Frontiers Science Research Base of Exercise and Metabolic Health, Shanghai University of Sport, Shanghai, China

**Keywords:** serine protease inhibitors, cathepsin G, thrombin, Cardiometabolic diseases, inflammation, obesity, insulin resistance, NAFLD

## Abstract

Murine Serpina3c belongs to the family of serine protease inhibitors (Serpins), clade “A” and its human homologue is SerpinA3. Serpina3c is involved in some physiological processes, including insulin secretion and adipogenesis. In the pathophysiological process, the deletion of Serpina3c leads to more severe metabolic disorders, such as aggravated non-alcoholic fatty liver disease (NAFLD), insulin resistance and obesity. In addition, Serpina3c can improve atherosclerosis and regulate cardiac remodeling after myocardial infarction. Many of these processes are directly or indirectly mediated by its inhibition of serine protease activity. Although its function has not been fully revealed, recent studies have shown its potential research value. Here, we aimed to summarize recent studies to provide a clearer view of the biological roles and the underlying mechanisms of Serpina3c.

## 1 Introduction

Murine Serpina3c, whose full name is serine (or cysteine) peptidase inhibitor, clade A, member 3C, consists of 417 amino acids with a molecular weight of 46~54KDa and is a serine protease inhibitor (Serpin). Its gene is located on chromosome 12 of mice. And the human homolog of Serpina3c is SerpinA3 (1, 2). In mice, Serpina3c is a secretory protein located outside the cells, which is mainly expressed in white adipose tissue (WAT), brown adipose tissue (BAT), heart, brain, pancreas and lung, but low in the liver, skeletal muscle, spleen, and kidney (3, 4). Recent studies have shown that Serpina3c is involved in some physiological processes, including insulin secretion, adipocyte differentiation, and also plays an important role in various pathologies (5, 6). However, the action mechanism of Serpina3c needs further study. The specificity and function of Serpin are defined by its reaction center loop (RCL), which extends from the main body of the peptide and guides the binding of inhibitors to target proteases (7). Under physiological conditions, Serpina3c, like its human homologue SerpinA3, can inhibit the activity of Cathepsin G and participate in the cascade of protein hydrolysis. In recent years, Serpina3c has been studied as a secretory factor of fat tissues in organisms. Through transcriptome analysis, it is shown that Serpina3c is a key factor in the signal network during adipogenesis. Researchers confirmed *in vitro* that Serpina3c is necessary for extracellular signal-regulated kinase (ERK) activation and glycogen synthase kinase-3β (GSK-3β) nuclear translocation, both of which are necessary for CCAAT/enhancer-binding protein β (C/EBPβ) phosphorylation and adipogenesis (6). In addition, it has been shown that Serpina3c can inhibit the turnover of α5/β1 integrin-mediated by cathepsin G, and the integrity of α5/β1 integrin signal transduction activates protein kinase B (AKT) to reduce the phosphorylation of c-Jun N-terminal kinase (JNK), thus inhibiting adipose tissue inflammation induced by high-fat diet (HFD) feeding in mice (3). However, the role of Serpina3c as a secretory factor of fat has not been fully revealed. Therefore, this paper reviews the role and mechanism of Serpina3c in various physiological and pathological processes, compares and analyzes it with its human homologue SerpinA3. This contributes to a better understanding of the biological similarities between mouse Serpina3c and human SerpinA3, as well as their roles in diseases. These findings may provide a deeper understanding of the role and mechanism of the Serpin family in physiological and pathological processes.

## 2 Homology and molecule features of Serpina3c

**Figure 1:**
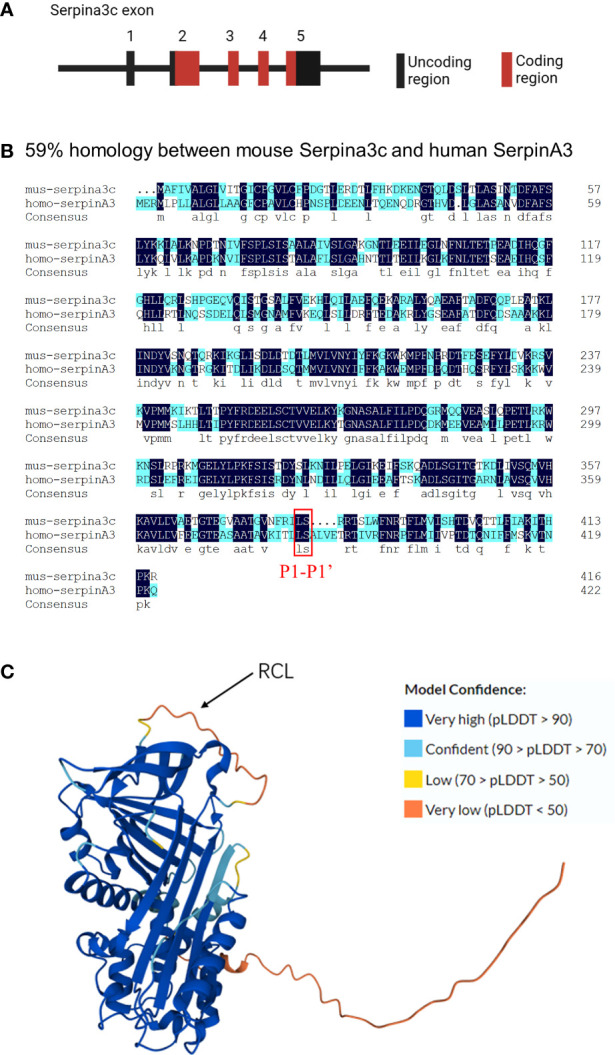
Molecular features of Serpina3c **(A)** The Serpina3c gene contains five exons, The first exon of the Serpina3c gene does not encode amino acid residues, and the second, third, fourth, and fifth exons of the Serpina3c gene encode 212, 92, 50 and 63 amino acid residues, respectively. **(B)** Comparison and alignment of murine Serpina3c protein sequences with human SerpinA3 protein. Two identical residues are represented in dark blue. A homology of 59% is detected between the murine Serpina3c protein and human SerpinA3 protein. The red box represents the P1-P1’ bond, which is conserved between Serpina3c and SerpinA3. **(C)** Prediction 3D structure of Serpina3c protein. The amino acid sequences were derived from UniProt (https://www.uniprot.org/uniprotkb/P29621), and the 3D structure was predicted by AlphaFold (https://alphafold.ebi.ac.uk/entry/P29621). AlphaFold produces a per-residue confidence score (pLDDT) between 0 and 100. Some regions below 50 pLDDT may be unstructured in isolation.

When attempting to isolate α-1 antitrypsin (SerpinA1) from mouse serum, researchers discovered two active ingredients, one of which was tentatively named Contrapsin (8, 9). Hill et al. found that murine Contrapsin shares homology with human α-1 antichymotrypsin (SerpinA3), a member of the clustered multigene family Spi-2 (10). Subsequently, mouse genes obtained from Mouse Genome Sequencing Consortium showed that a total 14 members of the Spi-2 gene family were located at murine chromosome 12F1, and a high degree of sequence homology indicated that these genes were all derived from the common ancestor of humans, namely *SerpinA3*, a single-copy gene on human chromosome 14q32.1. And *Serpina3c* is one of 14 members of the Spi-2 gene family (2). The first exon of the *Serpina3c* gene does not encode amino acid sequence, and the second, third, fourth, and fifth exons of the *Serpina3c* gene encode 212, 92, 50 and 63 amino acid residues, respectively (**Figure 1A**). Serpina3c and SerpinA3 share a high degree of sequence and structural similarity. According to the NCBI HomoloGene database (https://www.ncbi.nlm.nih.gov/homologene/111129), the nucleotide homology between Serpina3c and SERPINA3 is approximately 57%, while the protein homology is approximately 59% (**Figure 1B**), and both belong to the serpin family of proteins. In addition to their structural similarity, Serpina3c and SerpinA3 also have similar biological functions. For example, both proteins have been shown to play roles in blunting inflammation by inhibiting the activity of Cathepsin G (3, 11). Serpina3c and SerpinA3 can alleviate inflammatory response and prevent abnormal blood clotting by inhibiting the activity of serine proteases (12). Therefore, SerpinA3 is the human homologue of mouse Serpina3c.

Serpins have a highly conserved secondary structure, consisting of three β-sheets (A, B, and C), seven to nine α-helices, and a reaction center loop (RCL), which consists of about 17 amino acids connected to A and C β-sheets (7, 13). The Serpina3c protein consists of 417 amino acids, of which 1-22 is its potential signal peptide and 23-417 is its main chain. It can be glycosylated on its 38, 104, 184, 269 and 390 amino acid residues, but the effect of glycosylation on Serpina3c function is not clear. Its RCL is 367-392 of the amino acid sequence. The 3D protein structure prediction diagram of Serpina3c was generated by AlphaFold (14, 15) (**Figure 1C**). The specificity and function of Serpins are defined by their RCL, which extends outward from the main body of the protein and guides the binding with the target protease (16). RCL also acts as a pseudosubstrate and cleavage site for the cognate protease, and is the most variable portion of the molecule. The RCL residue is numbered relative to the cleavage point of the protease, which is defined as the P1-P1’ bond. The N-terminal residue of the cleavage point is P residue (P1, P2… Pn, moving away from the cleavage point), and the C-terminal residue of the cleavage point is P’ residue (P1’, P2’… Pn’) (17). Because the residues in the RCL match amino acids in the active site of the protease, it determines which proteases can be recognized, and the most critical residue is called P1, which determines the specificity of the RCL. P1 is flanked by a recognition residue called P4-P4’, which contributes to protease recognition by enhancing the affinity of the interaction (18, 19). The reaction site of protease in RCL cleaves the serine protease inhibitor and establishes a covalent bond between the reaction site of serine protease inhibitor and protease. The inactive serine protease inhibitor complex was highly stable and made serine protease inactive. Thus, the Serpins inhibition mechanism is “suicide,” and each serpin molecule is “single-use.” (20) Serpina3c has leucine at the P1 position flanked by phenylalanine and threonine at P4-P4’. The scissile bond is formed between leucine and serine at position P1-P1’, which is consistent with the structure of SerpinA3 at this site (21)(**Figure 1B**). This determines that the target protease of Serpina3c has a high degree of consistency with SerpinA3. Therefore, based on the molecular features, murine Serpina3c has a high homology to human SerpinA3.

## 3 Regulation of Serpina3c expression

**Figure 2:**
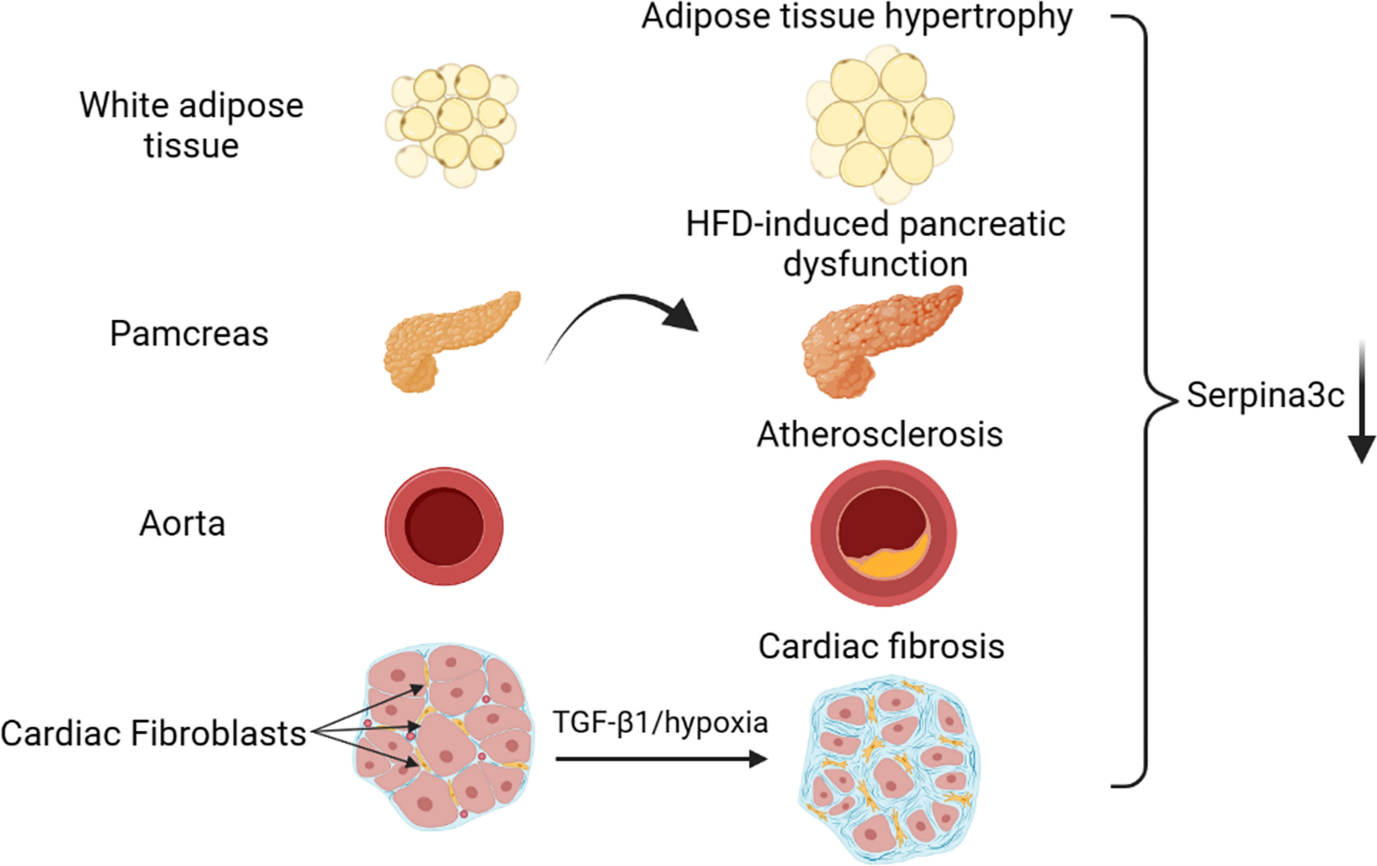
Serpina3c expression change in different tissues under cardiometabolic stress. Serpina3c protein expression is down-regulated in white adipose tissue (WAT) of obese mice induced by high-fat diet (HFD) feeding. Serpina3c protein levels were reduced in the pancreas of HFD-fed or Apoe^-/-^ mice compared with normal mice. Compared with normal mice, the mRNA and protein levels of Serpina3c in the aorta of mice in the Apoe^-/-^ group were down-regulated; and the mRNA and protein levels of Serpina3c were further down-regulated in Apoe^-/-^ mice fed with HFD compared with the ones fed with chow diet (CD). Serpina3c protein expression is down-regulated in primary mouse cardiac fibroblasts treated with transforming growth factor-β1(TGF-β1) or hypoxia for 24 h. (Created with BioRender.com).

There are few reports on the regulatory mechanism of Serpina3c expression. In the existing studies, we can find that the protein level of Serpina3c is down-regulated in adipose tissue, pancreas and blood vessels during obesity, pancreatic dysfunction and atherosclerosis induced by HFD feeding in mice (3, 5, 22).In addition, the protein level of Serpina3c was also down-regulated during the cardiac fibrosis induced by transforming growth factor-β (TGF-β) or hypoxia in mice (23) (**Figure 2**). However, the regulatory mechanism of Serpina3c expression has not been reported. We can refer to the regulatory mechanism of its human homolog SerpinA3.

It has been reported that SerpinA3 is one of the proteins secreted by the liver in response to acute inflammation, which is stimulated by tumor necrosis factor-α (TNF-α), interleukin-1β(IL-1β), and interleukin-6 (IL-6) (24, 25). Recombinant IL-6 (rhIL-6) treatment can stimulate SerpinA3 synthesis in human hepatoma cell lines. It is known that IL-6 can activate the janus kinase (JAK)/signal transducer and activator of transcription 3 (STAT3) pathway, and SerpinA3 has been identified as a direct transcriptional target of STAT3. Therefore, IL-6/STAT3 axis may regulate SerpinA3 during inflammatory responses (26). There is a TNF-α/IL-1 response enhancer in the distal enhancer region of -13kb upstream of the transcriptional initiation site of the SerpinA3 gene, which contains three elements: two binds to nuclear factor-kappa B (NF-κB) and one bind to activator protein 1 (AP-1) (27). IL-1β and TNF-α induce the expression of the SerpinA3 gene by activating NF-κB. However, inhibitor of NF-κB (IκB) could inhibit the activation of SerpinA3 by IL-1β and TNF-α. TGF-β can induce the expression of SerpinA3 in hepatocyte lines in synergy with IL-6 or TNF-α (28, 29). In addition, transcription factors (TFs) such as hepatocyte nuclear factor 4 (HNF4), zinc-finger TF ZBTB7B, Sp1 transcription factor (SP1), and myeloid zinc finger 1(MZF1) have also been reported to induce SerpinA3 expression (30, 31). At the same time, previous studies have shown that SerpinA3 is a target gene of nuclear receptor subfamily 4 group A member 1 (NR4A1). NR4A1 usually plays a role by binding with the NR4A1 response element (NBRE) in the promoter region of its target gene. The NBRE in the promoter region of SerpinA3 (-182 to -175) is a NR4A1-dependent functional DNA sequence. And subsequent NR4A1 overexpression and siRNA interference-mediated *NR4A1* gene knockdown analysis verified that SerpinA3 is indeed regulated by NR4A1 (32).

Elucidating the regulatory mechanism of SerpinA3 expression may play a guiding role in the study of the regulatory mechanism of Serpina3c expression, and may help better understand the regulatory networks and functions of Serpina3c in physiology and pathology.

## 4 Molecular function of Serpina3c

Serpins represent the largest and most versatile family of protease inhibitors, and its name comes from the function that the family was first described, which is serine protease inhibitor. Most serine protease inhibitors inhibit serine proteases (such as chymotrypsin, trypsin, and elastase), thus inhibiting the cascade of proteolysis and participating in physiological processes such as coagulation, inflammation, complement activation, and fibrinolysis (20, 33, 34). At present, it has been shown that the molecular function of Serpina3c is to act as a serine protease inhibitor, and is capable of binding to and inhibiting the activity of a wide range of serine proteases, including Cathepsin G and thrombin. In addition to its role as a protease inhibitor, Serpina3c has also been suggested to have other functions, such as playing a role in regulation of the secretory function of the pancreas and promotion of adipogenesis. Similarly, its human homolog SerpinA3 can also inhibit various serine proteases, including chymotrypsin, Cathepsin G, mast cell chymase, human glandular kallikrein 2, kallikrein 3, and lung serum proteases (35). Among these proteases, Cathepsin G is the main target of mouse Serpina3c and human SerpinA3. In addition, a special feature of SerpinA3 is that it can bind to DNA, but the physiological significance of this binding is not clear (36). Through the above targets, Serpina3c/SerpinA3 is involved in various biological processes such as cell proliferation, cell death, inflammation and so on.

Serpins exert its inhibitory effect by interacting with protease in two ways: inhibition pathway and substrate pathway, which are not mutually exclusive, and lead to protease inhibition through suicide mechanism (7). Thus, Serpins inhibits protease to make it lose its function through irreversible structural changes. In the inhibitory pathway, RCL binds to the protease, breaks at the P1-P1’ bond, and then slides into the A-β sheet, bringing the protease into the bottom of the Serpin protein to form a covalent complex, which irreversibly connects the Serpins with the protease, making the protease lose its solubility. In the substrate pathway, Serpin acts as the substrate of protease, and its structure is not modified and binds to protease, which hinders its function. Some Serpins require the presence of cofactors to function and are activated when and where they are needed. For example, heparin is a cofactor of SerpinA4 (37). SerpinA3, like other serpins, can inhibit proteases through both the inhibition pathway and substrate pathway. These two pathways are not mutually exclusive and can lead to protease inhibition through a suicide mechanism. The specific mechanism of protease inhibition for SerpinA3 may vary depending on the protease being targeted. The current research has not yet confirmed which way Serpina3c exerts its inhibitory function, and whether there is a cofactor for Serpina3c. We speculate that Serpina3c, like SerpinA3, can exert its inhibitory effect through both the inhibition pathway and substrate pathway. However, further research is needed to determine the specific mechanism of Serpina3c-mediated inhibition of target proteases.

## 5 Physiological roles of Serpina3c

### 5.1 Insulin secretion

**Figure 3:**
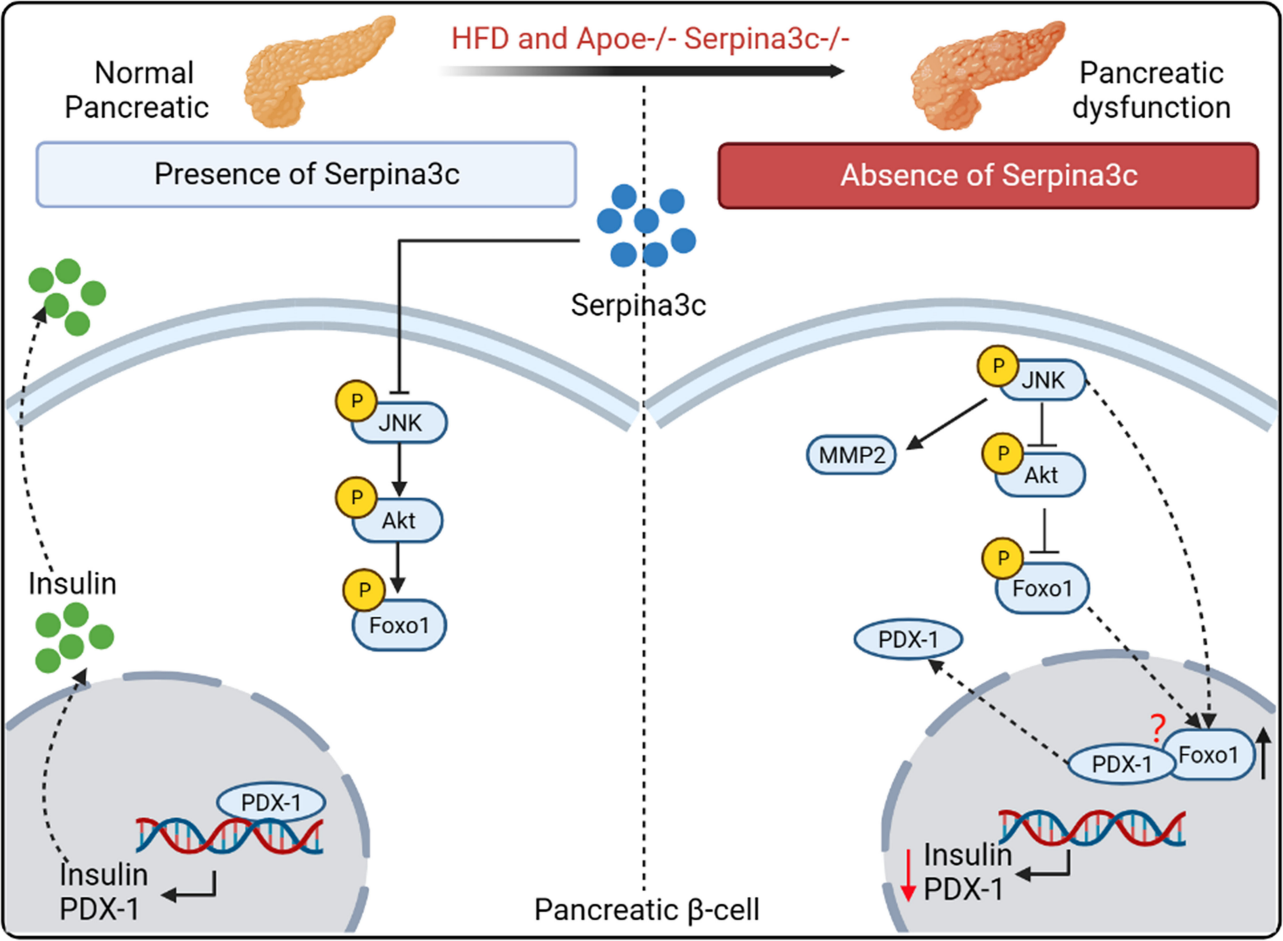
The role and mechanism of Serpina3c in the pancreatic β cells. Serpina3c can protect against pancreatic dysfunction induced by HFD feeding. Under HFD feeding in Apoe^-/-^ mice, the expression of phosphorylated JNK in the pancreas increased, which inhibited the phosphorylation of AKT, thus reducing the inhibition of phosphorylation of forkhead box protein O1 (Foxo1) and increasing the entry of Foxo1 into the nucleus. Overexpression of JNK can also promote the entry of Foxo1 into the nucleus. Foxo1 inhibits the activity of pancreatic transcription factor pancreatic and duodenal homeobox 1 (PDX-1) and the transcription of insulin and PDX-1 genes in the nucleus. And Foxo-1 is involved in the nucleocytoplasmic translocation of PDX-1, which promotes the transfer of PDX-1 from the nucleus to cytoplasm. However, the mechanism of Foxo-1-mediated nucleocytoplasmic translocation of PDX-1 needs to be further studied. Serpina3c can facilitate insulin secretion by inhibiting JNK/AKT/Foxo1/PDX-1 signal pathway in the pancreatic β cells of HFD-fed Apoe^-/-^ mice. (Created with BioRender.com).

The pancreas plays a key role in regulating metabolic/energy homeostasis through the release of various digestive enzymes and pancreatic hormones (38). In prediabetes, insulin secretion increases to compensate for the emergence of insulin resistance. HFD destroys normal pancreas structure and affects insulin sensitivity, thereby inducing prediabetes. The main stimulus for insulin secretion is the rise in blood glucose levels after a meal. In addition to glucose, free fatty acids (FFAs) can regulate insulin release through fatty acid metabolism. The researchers constructed Apoe and Serpina3c double-knockout mice to study the regulation of Serpina3c on insulin secretion from pancreatic β cells. Compared to Apoe^-/-^ mice, Apoe^-/-^Serpina3c^-/-^ mice had significantly higher blood lipid levels and higher serum FFAs after HFD feeding. Acute exposure to FFAs can stimulate insulin secretion, but when β cells are exposed to FFAs for a long time, it will reduce glucose-stimulated insulin secretion and damage β-cell function (39). With the prolongation of HFD feeding, the insulin secretion of β-cells in the Apoe^-/-^Serpina3c^-/-^ mice increased at first and then decreased. This study confirmed for the first time that Serpina3c can regulate insulin secretion of pancreatic β cells (5). At the same time, after Apoe^-/-^Serpina3c^-/-^ mice were fed with HFD, compared with Apoe^-/-^ mice, the level of phosphorylated AKT (p-AKT) and phosphorylated forkhead box protein O1 (p-Foxo1) in the pancreas decreased, and the expression of pancreatic and duodenal homeobox 1 (PDX-1) also decreased (5) (**Figure 3**). Previous studies have shown that PDX-1 can promote pancreatic β-cell proliferation and inhibit β-cell apoptosis (40). Moreover, PDX-1 promotes insulin secretion and maintains the normal function of pancreatic β-cells by regulating the expression of insulin and insulin-related genes such as *glucokinase* (*GCK*) and *solute carrier family 2 member 2* (*GLUT2*), etc (41).

If β cells were chronically exposed to a high-glucose and high-fat environment, the expression of the insulin gene decreased and was accompanied by a decrease in the expression of PDX-1 in the nucleus. When AKT signaling in β-cells is blocked, insulin secretion decreases under glucose stimulation (41, 42). Dephosphorylation of Foxo1 is increased upon inactivation of phosphatidylinositol 3-kinase (PI3K)/AKT signaling in pancreas. However, PDX-1 is a canonical promoter of insulin transcription, and its increased expression can be upregulated by increasing Foxo1 phosphorylation in pancreatic β-cells. In the absence of Serpina3c, β-cell function is inhibited due to the dysregulated AKT/Foxo1/PDX-1 signaling, leading to reduced insulin secretion. And the study also showed that compared with Apoe^-/-^ mice, Apoe^-/-^Serpina3c^-/-^ mice fed HFD had increased p-JNK levels, significantly increased matrix metallopeptidase 2 (MMP2) expression, and significantly decreased TIMP metallopeptidase inhibitor 2 (TIMP2) expression in the pancreas. JNK is a key molecule in the classic inflammatory signaling pathway. Their research also proved that Serpoina3c knockout can increase the number of F4/80 positive macrophages in the pancreas, and promote the levels of inflammation. JNK activation increases under oxidative stress and induces Foxo1 nuclear localization. Activated JNK pathway reduces Akt activity in β cell line HIT-T15 cells, resulting in reduced Foxo1 phosphorylation and its increased nuclear localization. Adenovirus-mediated overexpression of Foxo1 can reduce the nuclear expression of PDX-1, leading to pancreatic cell dysfunction (40, 43). This study also showed that Foxo-1 promotes PDX-1 nucleocytoplasmic translocation mediated by JNK pathway. This is consistent with the results found in mouse β cells after Serpina3c knockout, so it can be speculated that Serpina3c may protect the function of β cells through inhibiting the JNK pathway under the condition of HFD feeding. Current studies have not confirmed whether overexpression of Serpina3c can protect β cell function, and what is the mechanism of Serpina3c-mediated inhibiton of JNK. The protective effect of Serpina3c on β cell function needs to be further studied. SerpinA3 also has a high expression level in the pancreas (12), but little is known about the function of SerpinA3 in the human pancreas. These findings of Serpina3c may help us to gain deeper insights into the role of SerpinA3 in the human pancreas and explore its role in pancreatic diseases. Therefore, research in Serpina3c in mice is crucial for understanding the role of SerpinA3 in the human pancreas.

### 5.2 Adipogenesis

**Figure 4:**
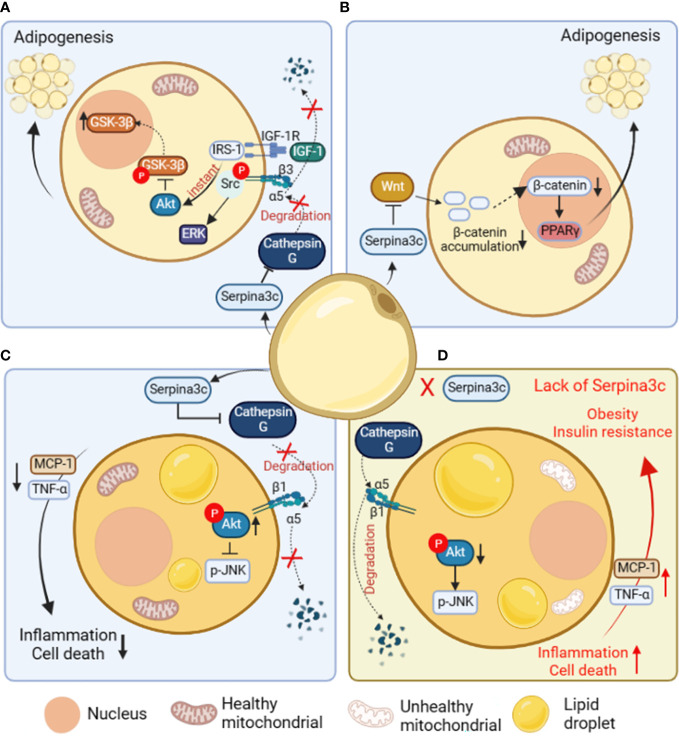
The role and mechanism of Serpina3c in preadipocytes and adipose tissue. **(A)** In preadipocytes, Serpina3c inhibits Cathepsin G, reduces the degradation of integrin α5, and promotes complex formation between insulin-like growth factors-1 receptor (IGF-1R) and integrin α5/β3 heterodimer. In the case of ligand binding, integrin β3 phosphorylates and binds to Src to activate extracellular regulated protein kinases (ERK). Insulin receptor substrate-1 (IRS-1) can instantly induce protein kinase B (AKT) signal transduction, reduce the phosphorylation of glycogen synthase kinase-3β (GSK-3β) to promote its nuclei entry, thereby facilitating adipogenesis. **(B)** In preadipocytes, Serpina3c can promote adipogenesis by inhibiting Wnt/β-catenin signal pathway, reducing the accumulation of β-catenin in the cytoplasm and its nuclei entry, and reducing the β-catenin-mediated transcriptional inhibition of peroxisome proliferators-activated receptor γ(PPARγ). **(C)** In adipocytes, Serpina3c maintained the integrity of integrin α5/β1 by inhibiting the activity of Cathepsin G and reducing the degradation of integrin α5. The increased integrity of integrin α5/β1 activates AKT, thereby reducing c-Jun N-terminal kinase (JNK) phosphorylation. This will lead to reduced release of inflammatory factors tumor necrosis factor-α (TNF-α) and macrophage chemoattractant protein-1 (MCP-1) from adipocytes, thereby inhibiting the inflammation of adipose tissue and cell death of adipocytes. **(D)** When Serpina3c is lacking in white adipose tissue, it cannot inhibit the degradation of integrin α5 by cathepsin G, resulting in a decrease in intracellular AKT phosphorylation and a failure to inhibit JNK phosphorylation, thereby promoting the release of inflammatory factors TNF-α and MCP-1 in adipose tissue, resulting in increased cell death. This eventually leads to insulin resistance and obesity in mice. (Created with BioRender.com).

Adipose tissue is a dynamic organ that plays an important role in physiological functions such as fat storage, energy homeostasis, and insulin sensitivity. Adipose tissue is divided into WAT, BAT and beige adipose tissue. Adipose tissue contains a variety of cell types, including mature adipocytes. White and brown adipocytes can be differentiated from different preadipocytes (44, 45). The complex molecular processes regulating adipocyte differentiation have not been fully determined (46). The current research has confirmed that the differentiation and generation of adipocytes is not only regulated by a variety of endocrine hormones, but also requires key transcription factors, including peroxisome proliferator-activated receptor γ (PPARγ) (47, 48), CCAAT/enhancer-binding proteins (C/EBPs) (49), signal transducers and activators of transcription (STATs) and Kruppel-like factor (KLF) proteins (50–52). 3T3-L1 is a preadipocyte line widely used in adipocyte differentiation research because the mature 3T3-L1 adipocytes are very similar to *in situ* adipocytes in morphology and biochemistry (53). It was shown that Serpina3c is a regulator of adipogenesis (4, 6). Serpina3c is a secretory protein that can reduce the degradation of integrin α5 by inhibiting Cathepsin G. Knockdown of Serpina3c interferes with insulin like growth factor 1 (IGF-1) signal transduction and disturbs the activation of ERK and AKT. The activation of the IGF-1 signaling pathway in 3T3-L1 cells requires the formation of a complex between the IGF-1 receptor and integrin α5-β3 heterodimer (53, 54). The binding of IGF-1 and its receptor can activate downstream targets such as ERK by phosphorylating integrin β3 to bind Src (55). The deletion of Serpina3c cannot prevent the degradation of integrin α5 by serine proteases such as cathepsin G and inhibit the activation of ERK mediated by Src. Due to the degradation of integrin α5, the integrin complex is absent, and IGF-1 signals through insulin receptor substrate 1 (IRS-1), resulting in sustained AKT signaling and the phosphorylation and nuclear export of GSK-3β (**Figure 4A**). Therefore, Serpina3c is necessary for ERK activation and GSK-3β nuclear translocation, while ERK activation and GSK-3β nuclear translocation are necessary for C/EBPβ phosphorylation and adipogenesis (4).

In the study of negative factors related to adipogenesis, it was shown that the Wnt signaling pathway inhibited adipogenesis by reducing the expression of PPARγ and C/EBPα in 3T3-L1 cells (56). Wnts signals are sent through Frizzled receptors and low-density lipoprotein receptor-related proteins in an autocrine or paracrine manner (57). When Wnt binds to its Frizzled receptor, it activates the typical pathway that inhibits GSK3, resulting in the increase of β-catenin in the cytoplasm, and then it translocates to the nucleus. β-catenin binds to T-cell factor/lymphoid enhancing factor(TCF/LEF) transcription factors in the nucleus to regulate the expression of Wnt target genes (58). Serpina3c knockout in female mice was associated with a reduction in the weight of visceral and subcutaneous adipose tissue, indicating impaired preadipocyte adipogenic differentiation capacity (6).. The impaired adipocyte differentiation ability is due to the increased expression of β-catenin in the Wnt signal pathway in visceral adipocytes after Serpina3c deletion. It was also confirmed in the 3T3-L1 cell line that after Serpina3c knockdown, the expression of β-catenin increased, and nuclear translocation occurred. It is proved that Serpina3c knockout may inhibit adipogenesis by activating the Wnt/β-catenin pathway to inhibit PPARγ (**Figure 4B**). SerpinA3 has also been reported to inhibit the Wnt signal pathway (12, 59), suggesting that SerpinA3 could also play a positive role in promoting adipogenesis. When it comes to the role of Serpina3c in adipogenesis, current research is mainly based on *in vitro* cell experiments, with a lack of *in vivo* studies. In addition, the regulatory effect of SerpinA3 on adipogenesis remains unclear, and further research is needed to confirm the precise role and mechanism of Serpina3c/SerpinA3 in adipocyte differentiation.

## 6 Pathophysiological roles of Serpina3c

### 6.1 Obesity and related metabolic disorders

There is growing evidence that obesity is associated with chronic inflammation, and inflammation in WAT promotes the development of morbid obesity and metabolic disorders (60, 61). In the case of excess calories, the expansion of adipose tissue can cause chronic inflammation and adipocyte death, and eventually lead to insulin resistance (62). Adipose tissue can secrete adipose factors and regulate adipose tissue and whole-body metabolism (63, 64). HFD-induced stress signals can affect the conversion rate and protein stability of some adipose factors, thus promoting diet-induced metabolic disorders (64). HFD decreased the expression of Serpina3c protein in mouse inguinal white adipose tissue (iWAT) and epididymal white adipose tissue (eWAT) (3). Of interest, a clinical study revealed that the serum levels of SerpinA3 were lower in prediabetic individuals with abdominal obesity when compared to non-obese prediabetic ones (65). The decrease of Serpina3c expression caused by HFD feeding further impairs insulin sensitivity and oxygen consumption in BAT, liver and muscle, and eventually led to obesity in mice. The study also showed that Serpina3c can improve insulin sensitivity and oxygen consumption of adipose tissue by inhibiting adipocyte inflammation (**Figure 4C**). Adipose tissue is an important metabolic tissue that regulates the dynamic balance of blood glucose. The lack of Serpina3c leads to the decrease of insulin sensitivity of adipose tissue, which further leads to the imbalance of glucose metabolism in the whole body, thus increasing the blood glucose intolerance (**Figure 4D**). Chronic low-grade inflammation caused by HFD feeding will affect the dynamic balance of lipid metabolism in adipose tissue, thus changing the content of lipids in circulation. A long-term increase in blood glucose levels and a combination of lipid metabolic disorders will lead to insulin resistance and systemic metabolic disorders. The local overexpression of Serpina3c in iWAT can promote the metabolic dynamic balance of adipose tissue by reducing the release of TNF-α and macrophage chemoattractant protein-1(MCP-1) to blunt inflammatory response of adipose tissue, thus improving the metabolic disorder related to obesity (3) (**Figure 4C**).

The researchers further explored the mechanism of Serpina3c regulating adipose tissue inflammation and found that Cathepsin G activity increased in adipose tissue of obese mice. Knockout of Serpina3c in adipocytes leads to increased Cathepsin G activity (4), and in Serpina3c knockout mice, adipose tissue inflammation increased with the increase of Cathepsin G activity. Overexpression of Serpina3c in iWAT decreased adipose tissue inflammation and inhibited Cathepsin G activity. This study shows that adipose tissue Serpina3c/Cathepsin G axis plays an important role in regulating adipose tissue inflammation and metabolic dynamic balance. The researchers then explored how Serpina3c regulates inflammation through Cathepsin G. They found that α5 and β1 integrin levels decreased when Serpina3c was deleted, but increased after overexpression of Serpina3c. Previous studies have shown that integrins are heterodimers containing transmembrane α and β subunits that play an important role in regulating development, immunity and inflammation (66). In the above study, integrin was found to be an intermediate molecule between extracellular Cathepsin G inhibition mediated by Serpina3c and intracellular AKT activation and JNK inhibition. Serpina3c inhibits Cathepsin G through its serine protease inhibitor activity, and maintains the integrity of α5/β1 integrin by inhibiting Cathepsin G. Then the increased integrity of α5/β1 integrin signal activates AKT and reduces the phosphorylation of JNK, thus inhibiting inflammation and promoting insulin sensitivity of adipocytes (3) (**Figure 4C**). Moreover, the expression of Serpina3c in mouse iWAT and eWAT was inhibited upon HFD feeding, and this inhibition occurred only at the protein level, not at the mRNA level. It is suggested that HFD feeding may inhibit the expression of Serpina3c through post-transcriptional action, such as promoting the degradation of Serpina3c protein, which needs further study. Because human SerpinA3 can also inhibit Cathepsin G (67), whether SerpinA3 can inhibit chronic adipose inflammation to ameliorate obesity and metabolic disorders by targeting Cathepsin G in humans merits further investigation.

### 6.2 Non-alcoholic fatty liver disease (NAFLD)

**Figure 5:**
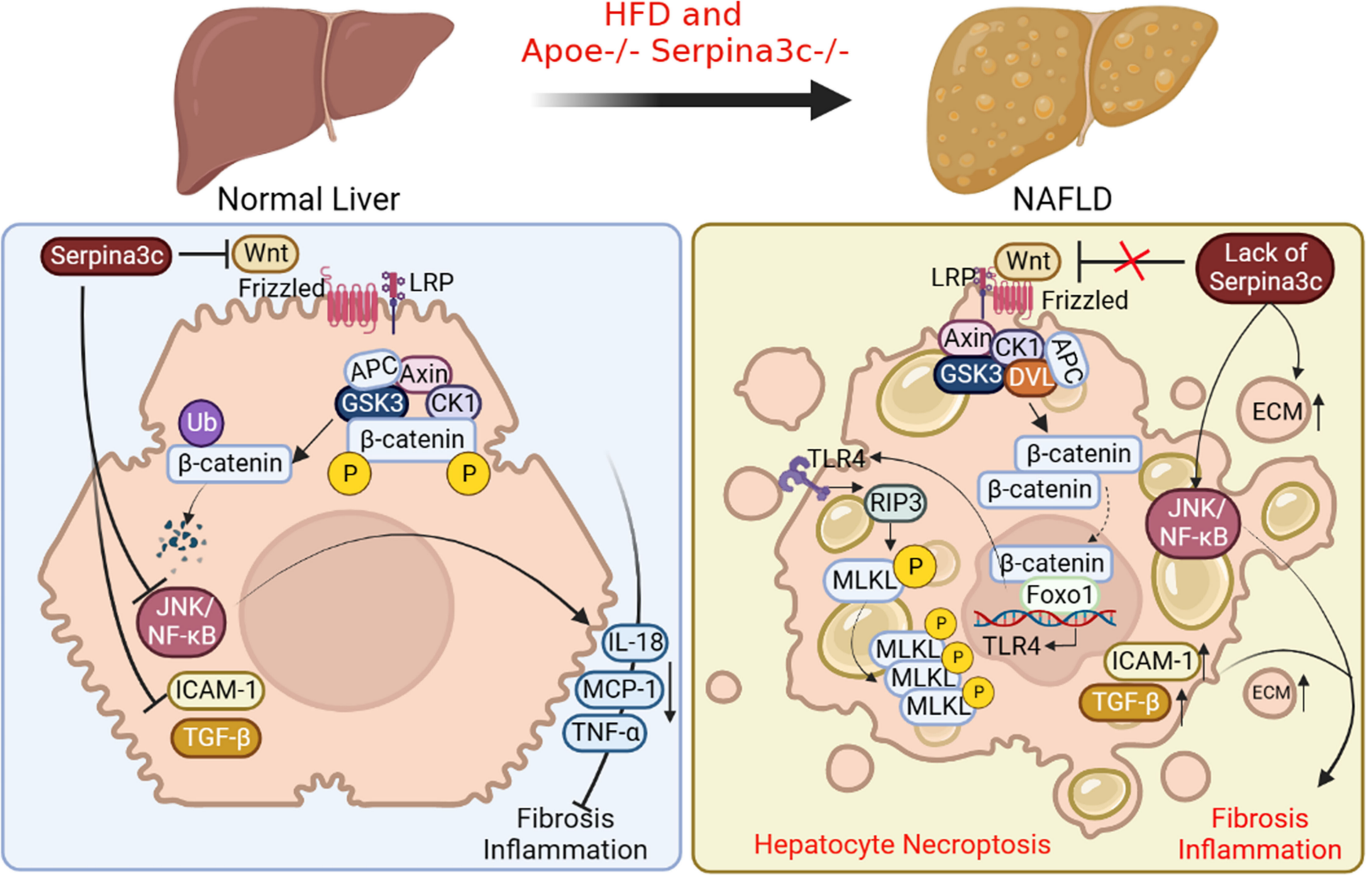
The beneficial roles of Serpina3c in NAFLD. In normal liver, Serpina3c can inhibit Wnt/β-catenin signal pathway, promotes ubiquitination-mediated degradation of β-catenin, inhibits JNK/NF-κB signal pathway, reduces the expression of inflammatory cytokines interleukin 18 (IL-18), MCP-1, TNF- α, and decreases the expression level of intercellular adhesion molecule 1(ICAM-1) and TGF-β. These will reduce liver inflammation and fibrosis. However, in the NAFLD livers of Apoe and Serpina3c double knockout mice fed with HFD, due to the lack of Serpina3c, Wnt/β-catenin signal pathway could not be inhibited, making the entry of β-catenin into the nuclei, which interacts with Foxo-1 to activate TLR4 transcription. Increased expression of TLR4, and activation of downstream receptor interaction protein 3 (RIP3) and phosphorylation mixed lineage kinase domain-like pseudokinase (MLKL) will then induce hepatocyte necroptosis. The deletion of Serpina3c leads to the activation of JNK/NF-κB signal pathway and the increase of the expression of ICAM-1 and TGF-β. The content of extracellular matrix (ECM) will be increased. Together, these lead to more severe inflammation and fibrosis in the liver. (Created with BioRender.com).

Nonalcoholic fatty liver disease (NAFLD) is the most common chronic liver disease characterized by excessive accumulation of triglycerides (TG) in hepatocytes without drinking alcohol (68–70). NAFLD includes simple steatosis to more serious diseases such as non-alcoholic steatohepatitis (NASH) (70, 71). NASH is characterized by steatosis, hepatocyte swelling, inflammation, and fibrosis (72, 73).And NASH can progress to cirrhosis, which is a liver disease characterized by fibrosis and structural remodeling due to chronic liver disease, leading to impaired liver function. Ultimately, hepatocellular carcinoma (HCC) may occur on the basis of cirrhosis (74, 75). Some studies have shown that compared with Apoe knockout group, Apoe and Serpina3c double knockout significantly promoted liver steatosis in HFD-fed mice, which further led to liver inflammation and liver fibrosis, and significantly aggravated liver injury (76). They found that Seprina3c deficiency also increased the expression of genes related to lipogenesis (*Srebf1* and *Scd1*) in the livers of HFD-fed mice, suggesting that Serpina3c may block the development of NAFLD. The accumulation of lipids and the increase of FFA levels in the liver lead to lipotoxicity and cell death in hepatocytes. More and more studies have shown that hepatocyte necroptosis plays a key role in promoting liver fibrosis and NAFLD progression (77). Receptor-interacting protein kinase 1 (RIPK1), RIPK3, and mixed pedigree kinase structure-like protein (MLKL) are the main molecules involved in necroptosis (78). RIPK1 recruits and phosphorylates RIPK3 to form a complex during necroptosis. The complex can then recruit and phosphorylate MLKL, leading to the oligomerization of p-MLKL. The translocation of p-MLKL to the plasma membrane causes an increase in membrane permeability and subsequent cell destruction by interacting with phosphatidylinositol (79). Many studies have proved that the typical RIP3-MLKL signal plays an important role in the pathogenesis of NASH (80–82). Serpina3c knockout promoted the expression of RIPK3 and p-MLKL in mouse liver. *In vitro*, Serpina3c inhibited palmitic acid (PA)-induced necroptosis (76), suggesting that Serpina3c can inhibit necroptosis under lipotoxic conditions, while Serpina3c deficiency increased the sensitivity of the liver to lipotoxicity and promoted necroptosis. Previous studies have shown that Toll-like receptor 4 (TLR4) can regulate necroptosis (83, 84). TLR4 can directly connect with RIP3 through TIR domain containing adaptor molecule 1 (TRIF) to activate and induce necroptosis (84, 85). TLR4 is the downstream target gene of transcription factor Foxo1, and its expression is regulated by Foxo1 (86). It has been shown that Serpina3c can inhibit the Wnt/β-catenin signal pathway and keep the intracellular β-catenin at a low level. The lack of Serpina3c makes the activated β-catenin enter the nucleus to bind to Foxo1 and activate it, and the activated Foxo1 increases the transcription of TLR4 (76). Therefore, as a Wnt inhibitor, Serpina3c can inhibit hepatocyte necroptosis by blunting the β-catenin/Foxo1/TLR4 pathway, which ameliorates NAFLD (**Figure 5**).

Many studies have confirmed that hepatocyte cell death can promote inflammation and fibrosis of NASH (87). JNK/NF-κB signal pathway is one of the important pathways related to the inflammatory response. It is reported that Serpina3c knockout greatly promotes the activation of JNK/NF-κB signal pathway and increases the expression of downstream target genes *TNF-α*, *IL-18*, and *C-C motif chemokine ligand 2 (CCL2)* in mice livers (76). In addition, Serpina3c knockout caused the expression of adhesion molecule intercellular adhesion molecule 1 (ICAM-1) in the liver (76), which indicated that Serpina3c knockout increased the infiltration of inflammatory cells in the liver. Fibrosis is another feature of NASH. Infiltrating inflammatory cells can release various cytokines and activate cells to secrete a large amount of extracellular matrix (ECM). It was found that the expression of TGF-β, the main regulator of fibrosis initiation, was up-regulated after Serpina3c knockout, which increased the deposition of collagen fibers in liver. Thus, Serpina3c deficiency promotes inflammation by activating JNK/NF-κB signaling pathway, leading to liver fibrosis. To sum up, Serpina3c can inhibit necrotizing apoptosis of hepatocytes, reduce liver inflammation and fibrosis, and prevent NAFLD from developing into more serious NASH (**Figure 5**). As its human homologue, SerpinA3 is mainly synthesized in the liver and secreted into the blood. However, at present, the research on the pathophysiological role of SerpinA3 in the liver is mainly focused on HCC. It has been found that the expression level of SerpinA3 decreases in the progression of liver cancer, and overexpression of SerpinA3 can inhibit the proliferation of liver cancer cells (88). And one study has pointed that SerpinA3 can inhibit the development and metastasis of liver cancer by targeting PTEN/PI3K/AKT/mTOR signal pathway (89). Currently the role of SerpinA3 in chronic liver disease is not well defined (90). Some studies suggested that SerpinA3 deficiency may be involved in the development and progression of liver fibrosis and cirrhosis (91) Further research is needed to better understand the mechanism of action of Serpina3c/SerpinA3 in chronic liver disease, including NAFLD.

### 6.3 Atherosclerosis of arteries

Atherosclerosis is characterized by chronic inflammation, the accumulation of lipids in plaques, and eventually leads to myocardial infarction (MI) and stroke (92). Abnormal proliferation and migration of vascular smooth muscle cells (VSMCs) is an important part of the pathological process of atherosclerosis. With the accumulation of inflammatory factors, growth factors, cytokines and vasoactive substances to the pathological level, the expression of cell proliferation-related proteins in VSMCs increased, resulting in excessive proliferation of VSMCs, then neointima and plaques are formed (92). Many studies have shown that thrombin plays an important role in atherosclerosis caused by abnormal proliferation and migration of VSMCs (93). It has been reported that some serpins can be used as thrombin inhibitors to inhibit blood coagulation (94). For example, both SerpinD1, SerpinC1 and SerpinE2 can participate in physiological processes such as blood coagulation, fibrinolysis and inflammation by inhibiting thrombin (13). Recently, researchers have found that the P1-P1’ of Serpina3c has the same amino acid sequence as SerpinD1, and they speculate that Serpina3c can be used as a thrombin inhibitor like SerpinD1 (22). Their conjecture was verified by protein-protein docking technique and co-immunoprecipitation, and confirmed that Serpina3c can interact with thrombin in the liver and aortic cusp.

**Figure 6:**
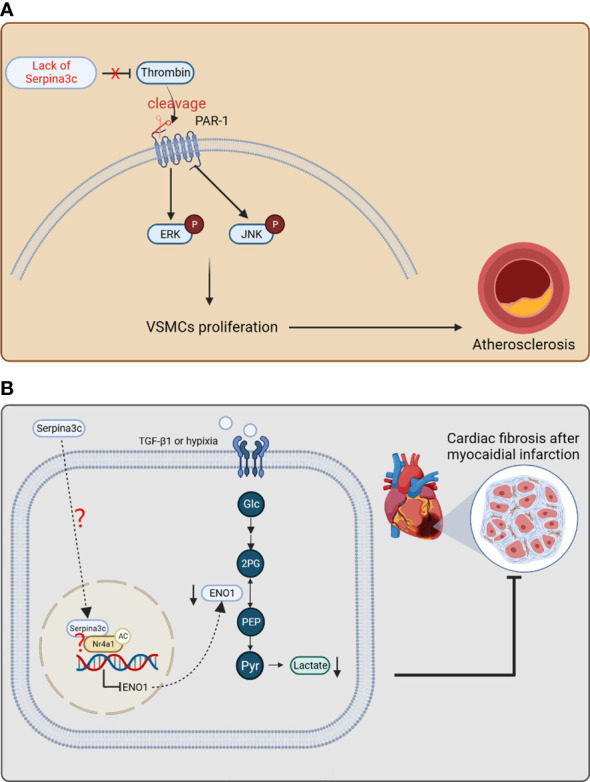
The protective effects of Serpina3c against atherosclerosis and myocardial infarction. **(A)** Serpina3c deficiency leads to the failure to inhibit the cleavage and activation of protease-activated receptor-1 (PAR-1) by thrombin. PAR-1 subsequently increases the phosphorylation of ERK and JNK, which promotes the excessive proliferation of arterial smooth muscle cells and leads to atherosclerosis. VSMCs, vascular smooth muscle cells. **(B)** In fibroblasts, Serpina3c enters the nuclei of mouse cardiac fibroblasts and interacts with Nr4a1 to promote Nr4a1 acetylation, which inhibits ENO1 transcription to prevent excessive activation of glycolysis. These will inhibit fibroblasts proliferation and differentiation, thus alleviating fibrosis after myocardial infarction. However, as a secretory protein, how serpina3c enters the nuclei and how it leads to acetylation of Nr4a1 remains to be studied further. (Glc, glucose; 2PG, 2-phosphoglycerate; PEP, phosphoenolpyruvate; Pyr, enolpyruvic acid.) (Created with BioRender.com).

The researchers then constructed Apoe and Serpina3c double knockout mice to study the role of Serpina3c in atherosclerosis. They found that the double knockout mice developed more severe atherosclerosis than the Apoe single knockout mice. Their results showed that the absence of Serpina3c led to increased neointima formation due to increased thrombin activation and elevated numbers of VSMCs and macrophages (22). Previous studies have shown that thrombin is a serine protease, which can promote cell proliferation and migration by activating protease-activated receptor (PARs) to trigger intracellular signaling pathways. Therefore, Serpina3c may regulate the proliferation of VSMCs through thrombin/PAR pathway. Next, they verified that Serpina3c reduces the activation of PAR-1 by inhibiting thrombin, thus inhibiting the proliferation of VSMCs. Thrombin can cleave the extracellular N-terminal domain of PAR-1, resulting in changes in its transmembrane domain and subsequent intracellular signal transduction. PAR-1 intracellular signal transduction can lead to thrombin-stimulated cell proliferation through a variety of kinases, including ERK1/2 and JNK kinases. In addition, the researchers found that ERK1/2 and JNK regulate the proliferation and migration of VSMCs, which are downstream signaling pathways of thrombin-PAR-1 axis. Serpina3c deletion stimulates the phosphorylation of ERK1/2 and JNK, but overexpression of Serpina3c in VSMCs inhibits the phosphorylation of ERK1/2 and JNK, and this inhibition is mediated by blocking the thrombin/PAR-1 axis. Therefore, Serpina3c can bind to thrombin and inhibit its activity, reduce the binding of thrombin to PAR-1, and inhibit the phosphorylation of intracellular signals ERK1/2 and JNK, thus reducing the abnormal proliferation and migration of VSMCs and blocking the progression of atherosclerosis (**Figure 6A**). At the same time, one study has shown that compared to wild-type mice, transgenic mice with human SerpinA3 overexpression had increased elastin content in the aorta, which may be associated with a more stable aortic phenotype. Additionally, a study revealed that the mRNA expression of SerpinA3 was reduced by 80% in biopsy samples of human abdominal aortic aneurysm (AAA) (95), suggesting that SerpinA3 plays a role in maintaining the structural and functional status of the arterial vessel wall. Overall, the studies on Serpina3c and SerpinA3 indicate that they may play protective roles against arterial diseases, and have significant implications for understanding the pathogenesis and treatment of arterial diseases.

### 6.4 Myocardial infarction

The chronic phase of myocardial infarction is characterized by cardiomyocyte hypertrophy and fibrosis remodeling (96). After moderate to severe myocardial infarction, glycolysis and fatty acid oxidation of cardiomyocytes were accelerated, and oxidative phosphorylation of glucose was inhibited (97). Due to myocardial ischemia, insufficient oxygen supply leads to an increase in the proportion of glycolysis, which will lead to excessive lactic acid production in the heart (98). Excessive lactic acid induces the proliferation and differentiation of myofibroblasts through pH- and TGF-β1 pathway, which leads to the aggravation of myocardial fibrosis (99, 100). In clinical practice, elevated circulating human SerpinA3 levels were significantly associated with adverse cardiovascular events after acute myocardial infarction (AMI), suggesting that human SerpinA3 may be a potential predictive marker for clinical manifestations of AMI (101). But the role of SerpinA3 upregulation in AMI needs further exploration. The researchers used a mouse model of myocardial infarction to explore the mechanism of Serpina3c in myocardial fibrosis after myocardial infarction (23). It was shown that the expression of Serpina3c protein was down-regulated in cardiac fibroblasts treated with TGF-β1/hypoxia. After the knockout of Serpina3c, the TGF-β1-induced proliferation ability of cardiac fibroblasts was significantly enhanced, which was reversed by the addition of Serpina3c recombinant protein. The myocardium of mice with Serpina3c knockout showed more severe myocardial fibrosis after myocardial infarction. These results indicate that Serpina3c plays an important role in curbing fibrosis after myocardial infarction.

Some studies have shown that the over-activation of glycolysis mediates the proliferation and differentiation of cardiac fibroblasts and participates in the process of fibrosis after myocardial infarction (102, 103). Enolase1 (ENO1) is a key enzyme in the glycolysis pathway, which catalyzes the reversible conversion of 2-phosphoglyceric acid to phosphoenolpyruvate during glycolysis. In addition, the expression of ENO1 in primary fibroblasts up-regulated the expression of pro-fibrotic genes, down-regulated the expression of MMP-1 and MMP-3 (104), and promoted the fibrosis phenotype *in vivo* and *in vitro*. After Serpina3c knockout, the ability of glycolysis and the expression of ENO1 increased in the myocardium of mice. After inhibition of ENO1, the enhancement of glycolysis caused by Serpina3c deletion was weakened and the myocardial glucose absorption decreased. Further study showed that Serpina3c can bind with Nr4a1 (also known as Nur77) in nucleus, which leads to activation of Nr4a1 and subsequent inhibition of ENO1 expression. In fibrotic disease, Nr4a1 deficiency leads to persistent activation of TGF-β signaling (105). It was shown that Serpina3c could bind to Nr4a1 in cardiac fibroblasts (CFs). The interaction of Nr4a1 and Serpina3c may inhibit the proliferation and differentiation of CFs. The regulation of Nr4a1 activity by Serpina3c may be through the regulation of its acetylation level. When it is acetylated, the protein stability of Nr4a1 is improved (106). This study confirmed that when Serpina3c was knocked out, the expression of histone deacetylase 1 (HDAC1) increased, meanwhile the acetylation level of Nr4a1 in CFs decreased. It was found that Serpina3c could promote the expression level and acetylation level of Nr4a1 in TGF-β1-treated CFs (23). In summary, Serpina3c inhibits the transcription of ENO1 in fibroblasts through Nr4a1 acetylation, inhibits the excessive activation of glycolysis and the proliferation and differentiation of fibroblasts, which improves cardiac remodeling after myocardial infarction (**Figure 6B**). A study showed that intravenous injection of recombinant human SerpinA3 before myocardial ischemia-reperfusion could reduce myocardial injury caused by myocardial ischemia-reperfusion in mice. It was pointed out that this protective effect was achieved by inhibiting the aggregation of neutrophils in the injured site (107). This study above, together with its mouse homologue Serpina3c studies, suggest that human SerpinA3 may have beneficial role in the protection against myocardial and injury, which is worthy of further investigation.

## 7 Conclusion and prospection

Murine Serpina3c is a member of serine protease inhibitor superfamily, clade “A”, mainly expressed in adipose tissue, pancreas, and the heart, but low in the lung, liver, skeletal muscle, spleen, and kidney. Because of its serine protease inhibitory activity, Serpina3c can target a variety of serine proteases, such as Cathepsin G and thrombin (3, 22), thus participating in a variety of biological processes. For example, Serpina3c can facilitate insulin secretion and promote pancreas function in high-fat diet feeding condition through inhibiting JNK-related pathways (5). As the murine homologue, human SerpinA3 is also highly expressed in pancreas, and its potential role in human pancreas deserves further studies. Serpina3c is also involved in adipogenesis which can inhibit Cathepsin G to regulate integrin/IGF-1-mediated ERK/AKT signal to promote adipogenesis (4). In addition, it can also promote adipogenesis and maintain normal adipose function by inhibiting Wnt/β-catenin pathway (6). Some studies reported that SerpinA3 can inhibit Wnt signal pathway by combining with LRP6 (59, 108). Thus, human SerpinA3 could also facilitate adipogenesis, which awaits further investigation. Under physiological conditions, the mechanism underlying the regulation of Serpina3c activity and expression is not clear, which is worthy of further exploration. Identifying the pathways and key factors that regulate the activity, expression, and stability of Serpina3c genes and proteins may help to better understand the physiological processes mediated by Serpina3c.

**Figure 7:**
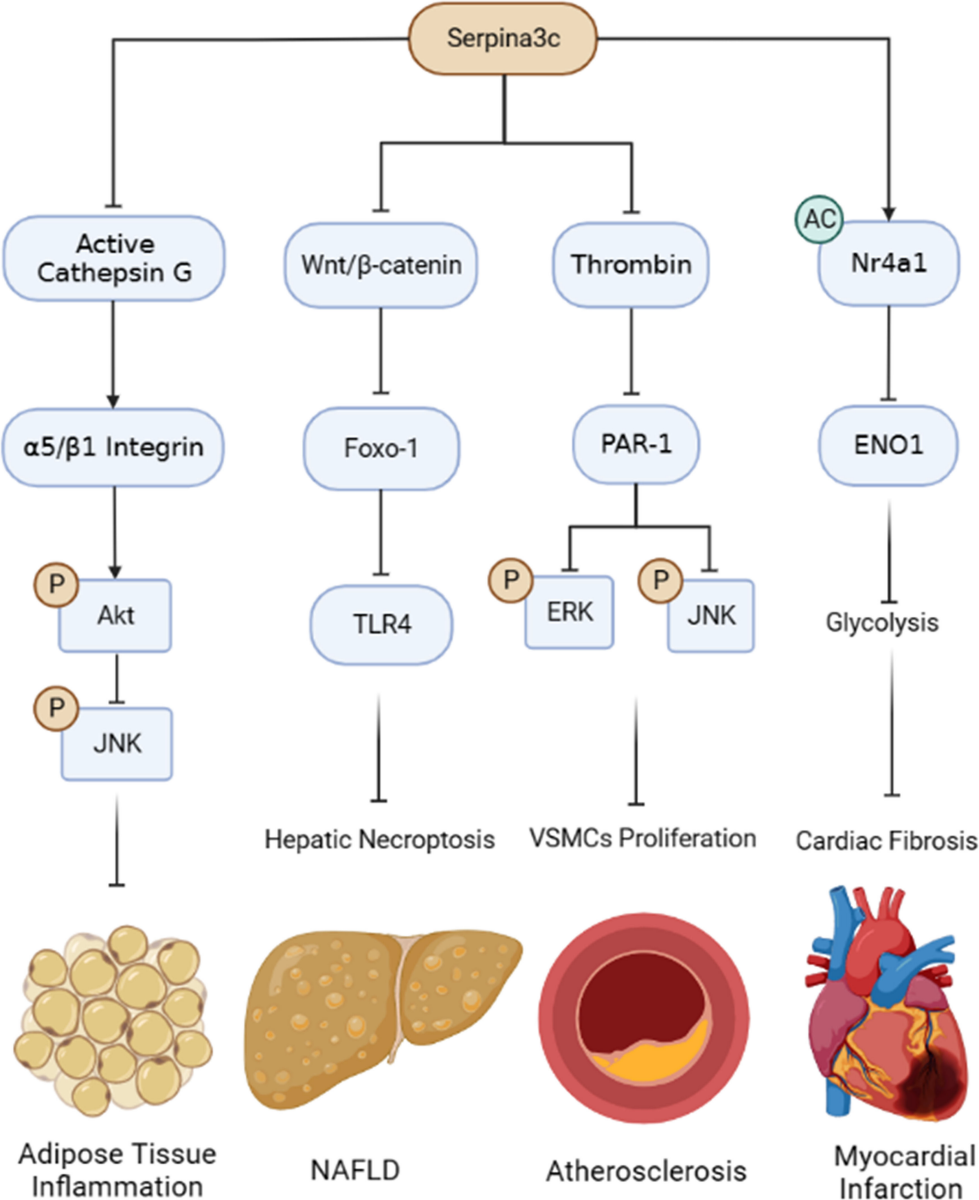
Summary of the protective roles and mechanisms of Serpina3c in pathophysiological processes. Serpina3c inhibits adipose tissue inflammation by inhibiting Cathepsin G, maintaining integrin α5/β1 integrity, activating AKT and inhibiting JNK phosphorylation. Serpina3c can inhibit non-alcoholic fatty liver disease (NAFLD) by inhibiting Wnt/β-catenin signal pathway in hepatocyte, reducing the transcription of Toll-like receptor 4 (TLR4) by Foxo-1, and inhibiting hepatic necroptosis. Seprina3c can bind and inhibit thrombin activity, prevent its cleavage and activation of protease-activated receptor-1 (PAR-1), weaken its downstream ERK and JNK phosphorylation, and inhibit the excessive proliferation of vascular smooth muscle cells (VSMCs), thus inhibiting atherosclerosis. Serpina3c can bind to nuclear receptor subfamily 4 group A member 1 (Nr4a1) in the mice cardiac fibroblasts nuclei and promote its acetylation, inhibits the transcription of enolase 1 (ENO1) and suppresses the excessive activation of glycolysis, thus alleviating cardiac fibrosis after myocardial infarction. (Created with BioRender.com).

Under pathophysiological conditions, the expression of Serpina3c protein in different tissues (adipose tissue, pancreas, aorta, liver) will be down-regulated under high-fat diet feeding or Apoe knockout condition, and its deficiency would result in a more serious disease phenotype in mice. However, the molecular mechanism leading to the down-regulation of Serpina3c protein is unclear. The absence of Serpina3c induces necroptosis of hepatocytes and promotes the progression of NAFLD (76). Because SerpinA3 is reported to inhibit the progression of fibrosis and HCC, the role of SerpinA3 in preventing the pathogenesis of chronic liver diseases, including NAFLD, merits in-depth studies. Besides NAFLD, Serpina3c deficiency may also lead to other metabolic disorders, such as increased obesity and insulin resistance. Serpina3c can improve the metabolic phenotype by inhibiting Cathepsin G to blunt adipose tissue inflammation (3). As a potent inhibitor of Cathepsin G, the role of SerpinA3 in ameliorating adipose tissue inflammation remains to be confirmed. In addition, lack of Serpina3c leads to more severe atherosclerosis. Serpina3c can inhibit the excessive proliferation of VSMCs and alleviate the progression of atherosclerosis by inhibiting thrombin and reducing the phosphorylation of ERK1/2 and JNK (22). Furthermore, Seprina3c can inhibit the proliferation of cardiac fibroblasts (CFs) by suppressing the transcription of ENO1which alleviates cardiac fibrosis after myocardial infarction (23). Similarly, SerpinA3 also appears to play a protective role in cardiovascular disease (107). In addition, how Serpina3c is transported into the nucleus in CFs and how Serpina3c mediates the acetylation of Nr4a1 remains to be determined. Interestingly, SerpinA3 was also found to enter the nucleus to function as a possible transcriptional regulator (88). In general, Serpina3c could have a protective role in multiple diseases (**Figure 7**). The mechanism underlying the different roles of Serpina3c in various diseases awaits to be further elucidated. Importantly, as a homologue of human SerpinA3, studying the functions of Serpina3c in different physiological and pathological conditions can help us better understand the functional role of SerpinA3 in various biological processes and diseases in humans.

## Author contributions

The search and collection of literatures was performed by YL and LG. The first draft of the manuscript was written by YL and LG co-wrote the manuscript. All authors commented on previous versions of the manuscript. All authors contributed to the article and approved the submitted version.

## Glossary

**Table:**

AKT	protein kinase B
AMI	acute myocardial infarction
AP-1	activator protein 1
BAT	brown adipose tissue
C/EBPs	CCAAT/enhancer-binding proteins
C/EBPβ	CCAAT/enhancer-binding protein β
CCL2	C-C motif chemokine ligand 2
CFs	cardiac fibroblasts
ECM	extracellular matrix
ENO1	Enolase1
ERK	extracellular signal-regulated kinase
eWAT	epididymal white adipose tissue
FFAs	free fatty acids
Foxo1	forkhead box protein O1
GCK	glucokinase
GLUT2	solute carrier family 2 member 2
GPCRs	G-protein coupled receptors
GSK-3β	glycogen synthase kinase-3β
HCC	hepatocellular carcinoma
HDAC1	histone deacetylase 1
HFD	high-fat diet
HNF4	hepatocyte nuclear factor 4
ICAM-1	intercellular adhesion molecule 1
IGF-1	insulin like growth factor 1
IL-1β	interleukin-1β
IL-6	interleukin-6
IRS-1	insulin receptor substrate 1
iWAT	inguinal white adipose tissue
IκB	inhibitor of NF-κB
JAK	janus kinase
JNK	c-Jun N-terminal kinase
KLF	Kruppel-like factor
LRP6	low-density lipoprotein receptor-related protein 6
MCP-1	macrophage chemoattractant protein-1.MI, myocardial infarction
MLKL	mixed pedigree kinase structure-like protein
MMP2	matrix metallopeptidase 2
MZF1	myeloid zinc finger 1
NAFLD	non-alcoholic fatty liver disease
NASH	non-alcoholic steatohepatitis
NF-κB	nuclear factor-kappa B
NR4A1	nuclear receptor subfamily 4 group A member 1
PA	Palmitic acid
TLR4	Toll-like receptor 4
PARs	protease-activated receptor
PDX-1	pancreatic and duodenal homeobox 1
PI3K	phosphatidylinositol 3-kinase
PPARγ	peroxisome proliferator-activated receptor γ
RCL	reaction center loop
rhIL-6	Recombinant IL-6
RIPK1	Receptor-interacting protein kinase 1
Serpina3c	serine (or cysteine) proteinase inhibitor, clade A, member 3C
Serpins	Serine protease inhibitors
SP1	Sp1 transcription factor
STAT3	signal transducer and activator of transcription 3
STATs	signal transducers and activators of transcription
TCF/LEF	T-cell factor/lymphoid enhancing factor
TFs	transcription factors
TG	triglycerides
TGF-β	transforming growth factor-β
TIMP2	TIMP metallopeptidase inhibitor 2
TNF-α	tumor necrosis factor-α
TRIF	TIR domain containing adaptor molecule 1
VAT	visceral adipose tissue
VEGF	vascular endothelial growth factor
VSMCs	vascular smooth muscle cells
WAT	white adipose tissue
Wnt	Wingless
